# Sarcopenia: A Contemporary Health Problem among Older Adult Populations

**DOI:** 10.3390/nu12051293

**Published:** 2020-05-01

**Authors:** Sousana K. Papadopoulou

**Affiliations:** Department of Nutritional Sciences and Dietetics, School of Health Sciences, International Hellenic University, 57001 Thessaloniki, Greece; souzpapa@gmail.com; Tel.: +30-6944798916

**Keywords:** sarcopenia, exercise, nutrition, supplements, older adults, muscle mass, frailty

## Abstract

Sarcopenia, a geriatric disease characterized by a progressive loss of skeletal muscle mass and loss of muscle function, constitutes a rising, often undiagnosed health problem. Its prevalence in the elderly population is largely considered variable, as it ranges from 5% to 50% depending on gender, age, pathological conditions as well as diagnostic criteria. There is no one unified approach of treatment or assessment, which makes sarcopenia even harder to assess. There is a pressing need to provide better diagnosis, diagnostics, prevention, and individualized health care. Physical activity and nutrition are the main studied ways to prevent sarcopenia, and they also offer better outcomes. This review aims to report the prevalence of sarcopenia in older adults, its etiology, prevention, and treatment techniques.

## 1. Introduction

Sarcopenia is predominantly a geriatric condition, with a gradual loss of skeletal muscle mass and a loss of muscle function [1], first described by Rosenberg [2]. It is one of the leading health issues in the older adults, and it increases disability risk, falls as well as injuries related to falls, hospitalization, limitation of independence, and mortality [3]. Risk factors for sarcopenia include age, gender, level of physical activity, and the presence of chronic disease as well as human immunodeficiency virus (HIV) [4,5,6,7]. Its incidence varies widely depending on the population surveyed (such as variations in sex, age, ethnicity, and body composition between various ethnic groups), living conditions (hospitalized, community dwelling, and nursing homes), and assessment tools and methods. In fact, there are several definitions for sarcopenia, with no consensus, hence its prevalence may vary widely [8]. This review aims to report the prevalence of sarcopenia within the older adult age group, its etiology, prevention, and treatment techniques.

## 2. Pathophysiology

Sarcopenia is a multifactorial disease [9], with a few of its identified contributing factors being low levels of physical activity—likely being a contribution to muscle mass decline—[10,11], decreased caloric intake [12], progressive increase in fibrosis, muscle metabolism changes, chronic inflammatory state, oxidative stress, and neuromuscular junction degeneration [13].

The cellular and molecular mechanisms behind sarcopenia are well described by Riuzzi et al. [14].

Low levels of physical activity are among the main risk factors for sarcopenia, along with the muscle fiber decline [15] that begins in midlife. A gradual loss of muscle fibers begins at 50 years and approximately 50% of the fibers are lost by the age of 80, while the muscle fiber loss is also seen in athletes [15].

In addition to this, hormonal changes with age in growth hormone, testosterone, thyroid hormone, and insulin-like growth factor lead to muscle mass and muscle strength loss, in conjunction with catabolic signals by tumor necrosis factor-α (TNF-α) and interleukin-6 (IL-6) [13], which are in imbalance with the anabolic signals [16]. Furthermore, inadequate nutrient intake and low protein synthesis are common in older adults, while a buildup of lipofuscin and cross-linked proteins in skeletal muscles has been proposed as a factor for low muscle strength in people with sarcopenia [17]. Moreover, another cause of sarcopenia that has been proposed is the failure of satellite cell activation in the muscle [13].

From a histological point of view, it has been found that the sarcopenic state affects the type II muscle fibers with the effect of decreasing their amount, their size, and the number of their mitochondria [18,19]. Among older adults in particular, food consumption has been recorded to be reduced by 25% [20], with quality of food intake, being significantly compromised [21]. Reduced protein intake and low vitamin D levels have also been found to correlate with the diminished muscle strength [10,22,23]. Hormonal decline associated with aging is also likely to impact the loss of muscle mass, with reduced amounts of testosterone and estrogen in men and women, respectively [23,24,25,26].

Chronic inflammation is a contributing factor to almost every known disease [27,28,29]. Aging is characterized by an increase in inflammatory markers and its related factors. Aging-related inflammation in the absence of infection is characterized as low-grade, chronic, and systemic, resulting in responses that contribute to degeneration of tissues. Aging-related inflammation is expected to result from a decreased immune response or lifelong exposure to antigenic stimuli [30,31], resulting in the development of reactive oxygen species and tissue damage via the release of cytokines mediated by the innate and acquired immune system [32]. In action, age-related inflammation is followed by age-related decrease in the number of T and B cells, along with a rise in natural killer cells [33], and tumor necrosis factor-α (TNF-α), interleukin-6 (IL-6), interleukin-1(IL-1), and C-reactive protein (CRP) [34,35]. Subsequently, it is proposed that such cytokines contribute to a predisposition to sarcopenia by triggering the ubiquitin–protease system [36,37]. This altered activation of the cell signaling pathway is known to promote the inflammatory state irrespective of tissue damage or antigenic exposure, further leading to one of the pathogenetic bases that underlie sarcopenia [38,39]. This state also leads to anabolic resistance, which is one of the major determinants of sarcopenia, suggesting that the skeletal muscle protein synthesis in response to physiological stimuli in the older population is below the level of muscle maintenance [40].

Furthermore, myostatin, a protein produced from and released by myocytes affects muscle cell function to inhibit myogenesis [41] by inducing the formation of the SMAD transcription altering protein complex (the main signal transducers for receptors of the transforming growth factor beta (TGF-β) superfamily, which are fundamentally important for adjusting cell development and growth) [42]. The effects of peroxisome proliferator-activated receptor-γ coactivator 1 α (PGC-1α), a transcriptional coactivator that enhances mitochondrial biogenesis as well as inhibits transcriptional activity of FoxO (a family of proteins crucial in regulating the expression of genes that play a role in cell growth, proliferation, differentiation, as well as longevity), are also suppressed by myostatin [42]. There is a correlation between elevated myostatin and reduced muscle mass in in both animal and human studies making it a potential mediator of sarcopenia as well as a therapeutic target [43,44,45].

Evidence shows that sarcopenia might be affected by a genetic predisposition. Large-scale genome-wide association studies evaluating the impact of genetic variation on gait speed, lean body mass, and grip strength discovered single nucleotide polymorphisms (SNPs) linked to synaptic function and neural maintenance, skeletal muscle fiber structure and function, and muscle metabolism [12].

There is also evidence connecting the molecular circadian rhythms with the maintenance of skeletal muscle. The circadian clock plays a critical role in many skeletal muscle physiological functions, and it is important to better understand the basic bio-physiological processes underlying those complex interactions. The significance of circadian expression for skeletal muscle structure, function, and metabolism becomes obvious when studying the muscle phenotype in models of molecular clock disruption. The loss of the *Bmal1* (brain and muscle Arnt-like protein 1) gene leading to sarcopenia and multiple pathological muscle disorders was observed to support this, including results such as decreased mitochondrial density and altered mitochondrial respiration, fiber-type changes, disrupted sarcomeric structure, and restricted function [46,47].

Epidemiological work into health and disease developmental origins has shown that early environmental effects on growth and development may have long-term impacts on human health [48]. Low birth weight is associated with decreased muscle mass and strength in adult life, a sign of a weak early climate [49,50]. One study showed that a substantial decrease in muscle fiber score is associated with lower birth weight, suggesting that developmental influences on muscle morphology may explain the association between low birth weight and sarcopenia [51].

## 3. Diagnosis

There are several diagnostic guidelines concerning sarcopenia. The major ones are the European Working Group on Sarcopenia in Older People (EWGSOP), the International Working Group on Sarcopenia (IWGS), the Asian Working Group for Sarcopenia (AWGS), and the American Foundation for the National Institutes of Health (FNIH) [52,53,54,55]. These guidelines suggest similar cutoffs for muscle mass, muscle strength, and physical performance for assessing and diagnosing sarcopenia [52].

In 2018, the Working Group (EWGSOP2) updated their initial definition of sarcopenia in order to take into account scientific and clinical evidence that came during the last 10 years. The new consensus (1) focuses on low muscle strength as a key characteristic of sarcopenia (cutoff points are: grip strength <27 kg for men and <16 kg for women and chair stand >15 s for five rises for both sexes), uses detection of low muscle quantity and to confirm the sarcopenia diagnosis (cutoff points are: appendicular skeletal muscle mass <20 kg for men and <15 kg for women), and identifies poor physical performance as indicative of severe sarcopenia (cutoff points are: gait speed ≤0.8 m/s); (2) updates the clinical algorithm that is utilized for sarcopenia case-finding, diagnosis and confirmation, and severity determination to (3) provide distinct cutoff points for measurements of indicators that identify and define sarcopenia [56].

The most accurate methods for assessing muscle mass in clinical settings are bioelectrical impedance analysis (BIA) and dual-energy X-ray absorptiometry (DXA), which is considered the gold standard, because of its accuracy, wide availability, and also because it is the only radiological tool with accepted cutoff values to diagnose sarcopenia [57,58]. There is evidence that measuring muscle mass through deuterated creatine (D3Cr) can reliably measure muscle mass otherwise obtained through DXA, and correlate better with physical activity [59,60]. In research settings, the EWGSOP2 advices the use of magnetic resonance imaging (MRI) and computed tomography (CT) as well as DXA [56].

Because of the variety of assessment techniques, cutoff points, and sarcopenia criteria, sarcopenia diagnosis can be difficult to understand. In addition, the significant variations in the prevalence of sarcopenia relative to the studied population (community dwelling, hospitalization, and living in nursing homes) make it much more difficult to develop preventive routines and therapeutic protocols and involve a more person-centered and focused approach [61].

## 4. Epidemiology and Prevalence

As mentioned above, a recent systematic review and meta-analysis with data from 35 articles and 58,404 individuals around the world estimated that the overall prevalence of sarcopenia was 10% both in men and women aged over 60 [62]. Table 1 details the results of several epidemiologic studies assessing the prevalence of sarcopenia using different methods.

In a systematic review and meta-analysis conducted by our laboratory in 2019 with data of 41 studies and a total of 34,955 participants, we concluded that the prevalence of sarcopenia in com-munity-dwelling individuals was 11% in men and 9% in women. The prevalence of sarcopenia in nursing-home older adults was 51% in men and 31% in women, whereas among hospitalized individuals it was found to be 23% and 24% for men and women, respectively [61].

An FNIH study of over 4900 patients ≥60 years old found the mean age of sarcopenic patients to be 70.5 years among males and 71.6 years among females [63]. Sarcopenia appears to be more common in individuals from non-Asian countries than in Asian individuals. Some of the main factors of this variability are body size, cultural background, ethnic characteristics, diet, and life quality. Moreover, the cutoff points for the Asian populations are lesser than those for the non-Asian individuals in both genders, having young people of the respective ethnic group as reference [11,62]. 

Moreover, it is recognized that the use of BIA as a way of calculating muscle mass underestimates fat mass and overestimates muscle mass [64]. The prevalence of sarcopenia based on the BIA in various studies was higher than that in the dual-energy X-ray absorptiometry (DXA)-based approach [65].

One systematic review and meta-analysis published in 2018 found that the prevalence of sarcopenia in nursing homes is 41% [66], which is four times higher than the prevalence in community dwelling individuals [62]. Table 1 shows the differences in the prevalence of sarcopenia according to the assessment method (DXA and BIA) and the population among various studies over the world. 

## 5. Management of Sarcopenia in Older People

Several studies indicate that muscle function in older sarcopenic adults can be balanced and even enhanced by physical exercise, as seen in Table 2, specifically resistance training. However, providing suitable exercises for bed-rested older adults is especially challenging due to underlying comorbidities, which restrict the availability of energy for exercise, and a restricted dietary regime of low-quality protein supply that exacerbates this issue further [22]. In contrast, community dwelling individuals are more physically active and have a better dietary regimen [79,80].

Sarcopenic individuals in nursing homes reported more sedentary activities, were less likely to report being currently physically active, and were also more likely to be malnourished [3,70,81].

Hence, the are additional risk factors for sarcopenia and functional decline in hospitalized individuals, because of reduced energy intake, low physical activity or prolonged bed-rest, depressed mood, and social isolation [82,83].

Community-dwelling individuals are more likely to be sarcopenic in situations where they are less physically active and lack good nutritional status [22,68,84,85,86]. Given the contemporary financial problems added to the age-related nutritional issues of older adults [21,87,88], attaining a good nutritional status becomes pivotal during older age.

### 5.1. Physical Activity and Exercise

#### 5.1.1. Resistance Training

The most studied method of exercise therapy is progressive resistance training (PRT) where participants exercise against an increasing load. As an established treatment for muscle atrophy, resistance training, is known to reduce hospital length of stay and increase muscle cross-sectional area and grip strength in older adult populations [89,90]. Hip replacement due to primary hip osteoarthrosis in patients aged 60–86 who did resistance training for the quadriceps muscle of the operated leg 2–3 times per week for 3–6 months had better outcomes than the standard rehabilitation regime patients [89]. A study by Liu et al. researching resistance training in adults with sarcopenia demonstrated the trouble that sarcopenic patients may have with resistance exercises by showing that there is no statistical difference in the Short Physical Performance Battery or 12 month gait pace relative to a non-resistance training control group [91].

Many studies have shown that isokinetic strength in an older adult population is improved by resistance training [92]. A large meta-analysis that investigated the relationship between physical exercise and muscle strength in a middle-aged population (40–65 years) has shown that resistance training has a greater impact on grip strength than other types of physical exercise [93].

Another plausible mode of resistance training is eccentric exercise. In this type of exercise, the muscle contracts while stretching itself (for instance during stairs descent). This form of muscle work has the advantage of increasing muscle strength with a reduced energy consumption and is suitable for energy-limited individuals due to the high-power and low-energy demands of eccentric contractions [94,95].

In their systematic review in 2019, Beckwée et al. stated that a high-intensity resistance training program is the best exercise for sarcopenic patients, but low-intensity resistance training may be sufficient to induce strength gains. Additionally, they recommend the following training parameters: 1–4 sets of 8–15 repetitions during 2–3 training moments a week [96].

#### 5.1.2. Aerobic Exercise

Aerobic exercise of the skeletal muscle induces adenosine triphosphate (ATP) production in the mitochondria and enhances aerobic ability, metabolic control, and cardiovascular function. It also contributes to the activation of mitochondrial biogenesis and dynamics and to the restoration of mitochondrial metabolism, decreases catabolic gene expression, and increases the synthesis of muscle proteins [97,98,99]. Various studies demonstrated that aerobic exercise controls myostatin expression in mRNA [100]. Given that age-related sarcopenia is associated with these molecular factors, aerobic exercise tends to have a protective effect. Harber et al. recorded that cycle exercise in both 20-year-old and 74-year-old subjects improved muscle size and strength [101]. In addition, Bori et al. confirmed that older subjects had improved mitochondrial biogenesis and mitochondrial fission protein (Fis 1) after 12 weeks of aerobic exercise training [102]. Collectively, aerobic exercise tends to ameliorate problems associated with mitochondria and improve muscle hypertrophy and strength.

#### 5.1.3. High-Intensity Interval Training (HIIT)

High-intensity interval training (HIIT) modalities provide intense cycles alternated with periods of reduced intensity for rehabilitation, offering physiological benefits in less time than conventional exercise regimens [103]. The physiological changes due to HIIT may be partly regulated by signaling pathways that are usually associated with endurance training. This is confirmed by the significant increase in the peroxisome proliferator-activated receptor-γ coactivator 1 α (PGC-1α; i.e., the master regulator of mitochondrial biogenesis in the muscle) following HIIT [104]. The increase in PGC-1α further highlights HIIT’s possible widespread health benefits given the positive effects of an increase in PGC-1α on oxidative efficiency, glucose absorption, antioxidant protection, and sarcopenia [104]. A study by Seldeen et al. showed that a 10 minute, 3 times a week, 4 month progressive HIIT protocol improves frailty status in aged mice with physical performance changes across multiple domains (strength, endurance, gait speed) [105]. A randomized controlled trial by Sculthorpe et al. investigating the efficacy of a low-frequency HIIT (LfHIIT) intervention on peak muscle power (peak power output (PPO)), body composition, and balance in lifelong sedentary but otherwise healthy males showed that 6 weeks of LfHIIT (one session every 5 days) were effective to induce clinically relevant improvements in absolute and relative PPO, but did not improve their static balance [106]. Moreover, when two maximal-intensity isokinetic eccentric resistance HIIT sessions were performed by aged individuals, they have shown that the elderly may participate in various physical activities, even high-intensity muscle-damaging activities, without a negative impact on muscle function and adaptation [107]. The previous finding is of importance, since it seems that eccentric training can induce health-promoting effects that may improve quality of life in elderly individuals [108].

#### 5.1.4. Multimodal Exercise

No particular form of exercise appears to sufficiently meet the criteria for therapeutic exercise in age-related sarcopenia and thus well-rounded aerobic and resistance exercise programs are recommended [109]. Multimodal exercise includes a mix of strength training, cycling, aerobic training, equilibrium training, and other activities. Lee et al. reported that 12 weeks of a circuit program improved walking and balance skills and muscle isokinetic functions [110]. Two systematic studies identified important effects of multimodal exercise programs on all sarcopenia subdimensions in stable older adults [111,112]. Furthermore, Liberman et al. also discussed the impact on vulnerable older adults and concluded that after various forms of workouts, both muscle strength and physical functionality can be enhanced [111].

#### 5.1.5. Whole-Body Vibration Therapy

In case of inability to exercise, passive exercise can be used in sarcopenia patients. Whole-body vibration therapy (WBV) in older adults has been shown to vastly improve various physical measures, including but not limited to leg isometric strength, knee dynamic strength, sit-to-stand performance, and jumping height [113]. Postmenopausal women saw increases in trunk and leg flexion strength only when combining strength and aerobic exercises with WBV [113]. Medium frequency and duration exercises appear most effective, with intervals at 40 Hz for 360 s significantly improving isokinetic knee extension in older adults [113,114]. In addition to this, a 12 week WBV in older adults can improve skeletal muscle mass, physical fitness, as well as quality of life [115]. However, long-term use of WBV is not suggested, as side effects such as spinal degeneration and increased serum testosterone and growth hormone can occur. Thus, safe protocols should be applied, when using WBV [116].

### 5.2. Diet and Supplementation

Although diet plays an important role on sarcopenia and its management, dietary interventions are not as well studied as resistance training’s proven role. As shown in Table 3, there is a large array of evidence that many dietary aspects may be important to the development of sarcopenia [71]. Food intake declines by about 25% from 40–70 years of age, and even more so when combined with a dietary pattern that could be characterized as monotonous, this may lead to insufficient nutrient intake. In sarcopenia, three areas were considered essential in terms of diet: vitamin D, calcium, and antioxidants.

Protein provides amino acids required for muscle synthesis. There is also evidence that the amino acid leucine may activate the signaling pathways leading to protein synthesis [71]. Considering leucine, findings in older people show that a high proportion of leucine was required in an essential amino acid mixture, in order to reverse the suboptimal muscle protein synthesis [121]. Skeletal muscle synthesis of aged mice was promoted by leucine-enriched whey protein supplements but not by isolated leucine [122]. In fact, for older men, 2.5 g of crystalline leucine co-ingestion with pure dietary protein could increase anabolic response [123]. In healthy older subjects, supplementation of β-hydroxy-β-methylbutyrate (HMB), a leucine metabolite, during 10 days of bed rest could preserve muscle mass [124]. However, a study in relatively young and healthy men (mean age 71) failed to show effect on strength or muscle mass, yet this may be due to the fact that the diet of the participants was low in leucine [71]. 

There is also a general concern that muscle production following a protein load may be blunted in older people [72], leading to the possibility that overall recommended protein intakes should be increased for older people. Observational evidence shows a strong correlation between protein intake and lean mass [72]. In aging, inflammation, and disease a higher dose of protein is needed to maximize muscle protein synthesis [125]. A dose of 1–1.2 g/kg body weight/day is considered to be optimal [126]. However, the literature suggests that protein consumption in amounts greater than the recommended daily allowance (RDA) can improve muscle strength and mass among older adults [127].

High-quality protein from whole foods, as well as dietary supplements providing isolated proteins, such as whey, casein, egg, meat, and soy increase accretion of postprandial protein and induce muscle protein synthesis [128]. 

In middle-aged and older adults, the impact of an egg-based and a cereal-based isocaloric and isonitrogenous breakfast was tested regarding whole-body protein synthesis, breakdown, and net balance. The egg-based breakfast intake resulted in a greater protein net balance, suppressing protein breakdown, while higher levels of essential amino-acids remained elevated, even after a standardized lunch, despite no difference in muscle protein synthesis [129].

Dairy is also a great protein source [130], and studies show a benefit in the elderly, concerning muscle mass and function. More to the point, the observational study by Radavelli-Bagatini [131,132] found a positive association between higher dairy intake and appendicular bone mineralization and muscle mass in elderly women, as well as greater whole-body lean mass and better physical performance. Dairy intake was measured via a food frequency questionnaire, body composition via dual-energy X-ray absorptiometry, and physical performance using hand-grip strength and timed up-and-go tests.

When comparing whey, casein, and casein hydrolysate, whey was found to be more effective in postprandial muscle protein accretion due to its faster digestion and absorption kinetics and its amino acid content and especially leucine [128]. Leucine ingestion can induce a reduction of urinary nitrogen loss. However, further research is needed [133,134]. Two studies from the same research group reported that, in young and older individuals, the ingestion of casein, which is slowly digested progressive or during sleep, intra-gastrically [135], could simulate muscle protein synthesis (MPS) nocturnally through hyperaminoacidemia and could even facilitate protein balance throughout post-exercise overnight recovery. Ingestion of 20 g of high-quality protein, equivalent to 20 g essential amino acids is optimal to stimulate rise in rates of MPS in older muscle [136].

A Cochrane review [72], however, found no clear impact of supplements on sarcopenia-related functional tests. Therefore, the quantity and composition of the dietary protein required to prevent and treat sarcopenia remains uncertain.

The timing of protein intake also plays a critical role in muscle synthesis, given the fact that, each meal allows accretion of protein for 2 to 4 hours. Increasing meal frequency and adequate evenly spaced protein intake across meals results in maximal protein anabolism during a long period of the day through postprandial protein accretion [136]. According to Mamerow [137], the 24 h muscle synthesis rate was 25% higher in healthy women and men when their protein intake was evenly distributed across meals, compared to isoenergetic and isonitrogenous diets with uneven protein distribution across meals. Protein-rich drinks consumed 2 hours after dinner, 30 minutes before sleep benefit MPS, muscle recovery, and overall metabolism both in the short and the long term. Furthermore, 30–40 g of casein protein ingested 30 minutes before sleep or via nasogastric tube increased overnight MPS in both young and older men, respectively [138].

The role of vitamin D in the pathophysiology of several diseases has also been highlighted lately [139]. For sarcopenia in particular, low vitamin D levels have been suggested to reduce skeletal muscle mass, leading to the development of sarcopenia [140]. More recently [141], fish intake has been reported to delay the onset of sarcopenia, due to its high protein, vitamin D and E, magnesium, and omega-3 content. Vitamin D polymorphisms were associated with muscle strength [142]; frailty (a disorder with some sarcopenia overlap) was also associated with vitamin D deficiency [143].

A study in women with post-stroke hemiplegia, who were followed for two years and were treated with vitamin D supplements, showed increases in type II muscle fibers and muscle strength as well as reduction in falls and hip fractures, in comparison to control group, suggesting improvements in atrophy [144]. Another systematic review and meta-analysis by Beaudart et al. studying various populations found improvements in muscle strength, particularly in ages >65 years old, with vitamin D supplementation of 400–4000 IU/day, for 1–60 months [145]. Muscle biopsies, however, could possibly determine more accurately the change in number and size of muscle fibers after vitamin D treatment.

Referring to protein supplementation, intervention studies of the impact of vitamin D on strength and physical performance have shown mixed results but, given that vitamin D deficiency is prevalent in the elderly, further studies are needed to determine its function in sarcopenia [146]. 

The cohort of InCHIANTI related the relationship between antioxidants in the composition of the body, with β-carotene and vitamin C being positively linked to the skeletal muscle mass [147]. β-carotene, especially, protects against the natural tendency to decrease in gait speed [148]. One of the best sources of omega-3 fatty acids is fatty fish. The Hertfordshire study found that grip strength improved by 0.48 kg in females and by 0.43 kg in males for every portion of fatty fish eaten. This outcome was further confirmed by an 8 week, randomized controlled trial of omega-3 supplementation in older adults, which recorded an increased rate of synthesis of muscle protein in the supplement group [149].

Concerning antioxidants, there is limited evidence for curcumin and bromelain’s role in decreasing inflammation [150,151]. In fact, curcumin supplementation, in the form of the bio-available Meriva supplement (1 tablet/day) added to a diet and exercise plan, can help improve strength and physical performance in healthy older adults with loss of strength and tiredness, either alone or in conjunction with other dietary supplements [152].

Furthermore, resveratrol in conjunction with exercise increased mitochondrial density and decreased muscle fatigue resistance in older subjects aged 65–80 years old. In fact, the authors stated that resveratrol could reverse sarcopenia better than exercise alone, by increased muscle fiber size and power [153]. 

The combination of dietary interventions and exercise may lead to better results in the management of sarcopenia [154].

### 5.3. Medication

Pharmacotherapy is a beneficial tool for treating sarcopenia for older people where exercise and dietary treatments are not effective methods of treating sarcopenia, where underlying co-morbidities can influence the supply of energy or digestive processes (e.g., in cancer cachexia). This is partly derived from the fact that a lot of older people may be unwilling or unable to do resistance training at the intensity required for it to be effective. 

Hormone administration is a particularly explored therapy for sarcopenia. Growth hormone has been shown to cause an increase in muscle mass [163]. Furthermore, its implementation has not explicitly altered functional results and is therefore of questionable value. This highlights the difficulties of choosing outcome measure(s) for sarcopenia trials [164]. Testosterone supplementation has been shown to increase both muscle mass and strength in men but is also related to adverse cardiovascular conditions [165]. 

The fresh field of concern is drugs influencing the renin–angiotensin system, and whether the respective drugs may have direct muscle effects. An observational study initially indicated that inhibitors of ACE (angiotensin converting enzyme) could be beneficial to physical function [166], this result was subsequently verified in a trial showing increased walking time of six minutes in those given perindopril [167]. A similar impact was not apparent in a spironolactone trial [168], and neither of these medications has yet demonstrated any benefit in terms of results more commonly associated with sarcopenia. 

Owing to their tissue selectivity, selective androgen receptor modulators (SARMs) are of particular interest. It is hoped that androgenic signaling will produce improvements in skeletal muscle mass and strength without dose-adverse effects with these drugs [169]. Myostatin, thalidomide, OHR/AVR118, celecoxib, VT-122, and anabolic agents such as ghrelin and its analogues, MT-102, BYM338, and ruxolitinib, are other compounds under investigation as sarcopenia therapies. In a phase 2 clinical trial for the treatment of cachexia in late-stage cancer patients, MT-102, the first-in-class anabolic catabolic transforming agent (ACTA), was recently tested. Test results indicated substantial increases in body weight in patients treated with 10 mg MT-102 twice daily over the test span of 16 weeks compared to substantial decreases in body weight in patients receiving placebo treatment [170]. MT-102 has been demonstrated to reverse sarcopenia in aged animal models [171]. More work is currently ongoing on MT-102 as a therapy for sarcopenia. 

To date, few sarcopenia pharmacotherapies have been attempted after phase 2 trials. Growth-promoting agents (including myostatin inhibitors, testosterone, and SARMs) have the potential to boost lean mass in older people but converting these benefits to clinically applicable muscle strength and physical activity changes needs further assessment [172]. 

The next generation of medications to enhance physical function would possibly specifically target muscle function, with little to no effect on muscle mass, which would match well with the sarcopenia-based diagnosis criteria based on strength and patient performance [173].

## 6. Impact on the Quality of Life

Sarcopenia is associated with multiple adverse outcomes such as comorbidities, physical disability, poor physical performance, depression, frequent falls and increased hospitalization, functional decline, and increased mortality [3]. As such, older sarcopenic individuals tend to experience a lower quality of life (QoL), mainly because of their decreased physical function ability. Clinicians must frequently provide older people with questionnaires assessing their QoL, in order to better understand their needs, prioritize their problems, facilitate patient–doctor communication and track changes, or response to the treatment received. Accordingly, there is an increasing need for the use of the appropriate QoL measures in clinical practice, so clinicians can focus on treating the patients and not the just the disease [174].

## 7. Conclusions

As the global population ages, the prevalence of muscle wastage relative to age will increase. Following a universal standardized diagnosis will assist the discovery of viable treatment options. Malnutrition and low physical activity seem to be the two major factors associated with sarcopenia. Individual targeted therapies including supplementation and diet could be very beneficial for sarcopenic individuals. However, there are no currently approved medical therapies for the treatment of sarcopenia. The increase of awareness and understanding of this disease is essential for the continued development of standardized treatment, as well as diagnostic options, which will in turn lead to better care and quality of life for our geriatric populations. An appropriate exercise regimen, accompanied by nutritional interventions should be of major importance for a better outcome of sarcopenic and geriatric patients.

## Figures and Tables

**Table 1:** Differences in the prevalence of sarcopenia according to the assessment method used and the population studied.

First Author	Country	Population	Criteria	Muscle Mass Assessment Method	Sample (N)	Prevalence (%, *n*)
Rossi [67]	IT	Community dwelling	EWGSOP	DXA	274	33%, *n* = 92
Silva Neto [68]	BR	Community dwelling	EWGSOP	DXA	70	10%, *n* = 7
Hai [69]	CN	Community dwelling	AWGS	BIA	834	11%, *n* = 88
Yu [22]	CN	Community dwelling	EWGSOP	DXA	4000	5%, *n* = 216
Dodds [70]	GB	Community dwelling	EWGSOP	BIA	719	21%, *n* = 149
Yang [55]	CN	Community dwelling	AWGS	BIA	384	16%, *n* = 61
Lera [54]	CL	Community dwelling	EWGSOP	DXA	1006	19%, *n* = 192
Zengin [71]	GM	Community dwelling	EWGSOP	DXA	486	12%, *n* = 59
Bianchi [72]	IT	Hospitalized	EWGSOP	BIA	655	35%, *n* = 227
Smoliner [73]	DE	Hospitalized	EWGSOP	BIA	198	25%, *n* = 50
Martone [74]	IT	Hospitalized	EWGSOP	BIA	394	15%, *n* = 58
Cerri [75]	IT	Hospitalized	EWGSOP	BIA	103	21%, *n* = 22
Buckinx [76]	BE	Nursing home	EWGSOP	BIA	662	38%, *n* = 252
Senior [3]	AU	Nursing home	EWGSOP	BIA	102	40%, *n* = 41
Liu [77]	CN	Community dwelling	AWGS	BIA	4500	19%, *n* = 869
Sobestiansky [78] ^n^	GB	Community dwelling		BIA	287	
1			EWGSOP			21%, *n* = 60
2			EWGSOP2			20%, *n* = 58
3			FNIH			8%, *n* = 24

AWGS, Asian Working Group for Sarcopenia; BIA, bioelectrical impedance analysis; DXA, dual-energy X-ray absorptiometry; EWGSOP, European Working Group on Sarcopenia in Older People. ^n^ Consists of different methods or definition for estimation of prevalence of sarcopenia.

**Table 2:** Impact of resistance training, aerobic training, and whole-body vibrational therapy in sarcopenia.

First Author	Population	Interventions	Regimen	Observed Outcomes
Suetta [89]	Older, hip-surgery patients	Resistance training	3 times/week, for 12 weeks	↓ hospital length of stay, ↑ muscle strength and muscle cross-sectional area compared to the controls
Hassan [90]	Nursing care facility residents	Resistance training	2 times/week, for 24 weeks	↑ grip strength versus control group
Geirsdottir et al. [117]	Elder adults	Resistance training	RE + LTPA: 12 weeks + 16–18 months	↑ Quadriceps strength (only RE) ↑ Timed up-and-go performance (RE + LTPA)
Liu [91]	Sarcopenic adults	Resistance training	3 times/week, for 1–8 weeks	No difference compared to the non-training controls at 12 months
Chen et al. [118]	Sarcopenic obese adults	Aerobic training	Dance, 60 min for 8 weeks	↑ Muscle mass ↑ Back extensor strength
Harber et al. [101]	Young and older men	Aerobic training	Cycle ergometer, 12 weeks, 20–45 min, 3–4 day/week, 60%–80% of HRR	↑ Quadriceps volume (−6%) ↑ Muscle size↑ Aerobic capacity
Lau [119]	Older adults	Whole-body vibrational therapy	1–7 sessions/week, for 6 weeks to 18 months	↑ muscle strength, improved jumping height and sit-to-stand performance
Candow [120]	Older adults	Resistance training with creatine supplementation	5–20 g/day, for 12–24 weeks	↑ muscle mass, chair rise performance, and knee extension strength

↑, increase; ↓, decrease; =, no change; HRR, heart rate reserve; RE, resistance exercise; LTPA, leisure-time physical activity.

**Table 3:** Impact of nutrition and supplementation in sarcopenia.

First Author	Population	Interventions	Treatment Duration	Observed Outcomes
Abe [155]	Older nursing home residents	Group 1: EAAs (3 g), vit-D (800 IU), medium-chain TGs (6 g); Group 2: EAAs (3 g), vit-D (800 IU), or long-chain TGs (6 g)	13 weeks	↑ muscle strength, ↑ walking speed
Bauer [156]	Older community-dwelling individuals	Whey protein (40 g), carbohydrates (18 g), fat (6 g), vit-D (1600 IU), and mixture of vitamins, minerals, and fibers	13 weeks	↑ lean mass, = muscle strength, = walking speed
Evans [157]	Older community-dwelling individuals	Group 1: Leucine (2 g), L-Carnitine (1.5 g), creatine monohydrates (3 g), Vit-D (400 IU); Group 2: L-Carnitine (1.5 g)	8 weeks	↑ lean mass (only in group 1), = muscle strength
Ispoglou [158]	Older community-dwelling individuals	Group 1: EAA mixture (15 g); Group 2: EAA mixture leucine-enriched (15 g)	13 weeks	↑ lean mass (only in group 2), = muscle strength
Leenders [159]	Diabetes mellitus type 2 older individuals	Leucine	24 weeks	= lean mass, = muscle strength
Verlaan [160]	Older community-dwelling individuals	Whey protein (20 g), Vit-D (800 IU)	13 weeks	↑ lean mass
Radavelli-Bagatini [132]	Older community-dwelling women	Group 1: Dairy (≥2.2 servings/day) Group 2: Dairy (≤1.5 servings/day)	3 months	In comparison with group 2, group 1 had: ↑ whole-body lean mass, ↑ ASMM, ↑ hand-grip strength
Alemán-Mateo [161]	Older healthy individuals	210 g of ricotta cheese/day	12 weeks	↑ ASMM
Dal Negro [162]	Older community-dwelling individuals. COPD patients.	EAA (8 g)	12 weeks	↑ muscle strength, = lean mass

↑, increase; ↓, decrease; =, no change; EAA, essential amino-acid; TG, triglyceride; COPD, chronic obstructive pulmonary disease; ASMM, appendicular skeletal muscle mass.
